# Characteristics of post‐stroke dysphagia: A retrospective study based on FEES

**DOI:** 10.1002/brb3.3161

**Published:** 2023-07-21

**Authors:** Fang Sun, Jia Qiao, Xiaoyan Huang, Zitong He, Zulin Dou

**Affiliations:** ^1^ Clinical Medical College of Acupuncture‐Moxibustion and Rehabilitation Guangzhou University of Chinese Medicine Guangzhou P. R. China; ^2^ Department of Rehabilitation Medicine People' Hospital of Yangjiang Guangzhou P. R. China; ^3^ Department of Rehabilitation Medicine Third Affiliated Hospital of Sun Yat‐sen University Guangzhou P. R. China

**Keywords:** fiberoptic endoscopic evaluation of swallowing (FEES), post‐stroke dysphagia (PSD), retrospective study, stroke lesion sites, tube feeding

## Abstract

**Objective:**

This study aims to examine the characteristics of dysphagia in stroke patients with different lesion sites and explore the factors that impact the duration of nasogastric tube after post‐stroke dysphagia (PSD).

**Methods:**

Patients with PSD were screened for analysis. Stroke types and lesion sites were confirmed using MRI or CT scans. Included patients were categorized into two groups: supratentorial stroke group (including lobar and deep intracerebral stroke subgroups) and infratentorial stroke group (including brainstem and cerebellar stroke subgroups). Fiberoptic endoscopic evaluation of swallowing (FEES), Penetration‐aspiration scale (PAS), Yale pharyngeal residue (PR) severity rating scale, Functional oral intake scale (FOIS), Murray secretion severity rating scale (MSS), laryngopharyngeal sensation, and vocal fold mobility were investigated to assess the swallowing function.

**Results:**

A total of 94 patients were included in the final analysis. Significant differences were found in PR scores (*p* < .001), PAS scores (*p* < .05), MSS scores (*p* < .05), and vocal fold mobility (*p* < .001) between infratentorial and supratentorial stroke groups. Moreover, lobar stroke showed significantly higher PR scores compared to the deep intracerebral stroke group (*p* < .05). Kaplan–Meier survival analysis indicated significant differences in the duration of nasogastric tube placement among the following groups: infratentorial versus supratentorial stroke, PAS ≤ 5 versus PAS > 5, PR ≥ 3 versus PR < 3, and normal vocal fold mobility versus vocal fold motion impairment group (*p* < .05).

**Conclusions:**

The infratentorial stroke may lead to worse swallowing function as compared to a supratentorial stroke. Additionally, patients with infratentorial stroke, PAS > 5, PR ≥ 3, or vocal fold motion impairment may contribute to a longer duration of nasogastric tube placement.

## Graphical abstract

The infratentorial stroke may lead to worse swallowing function as compared to a supratentorial stroke. Besides, infratentorial stroke, PAS > 5, PR ≥ 3, or vocal fold immobility may be related to the longer indwelling time of the gastric tube.

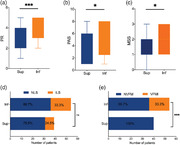

## 1 INTRODUCTION

Stroke is recognized as a leading cause of long‐term disability globally, significantly impacting patients' quality of life and imposing a substantial economic burden (Ding et al., 2022). Post‐stroke dysphagia (PSD) is a prevalent consequence of stroke, contributing to higher mortality and morbidity rates, with an estimated prevalence ranging from 30 to 80% (Banda et al., 2022; Chang et al., 2022; Ko et al., 2021; Takizawa et al., 2016). Individuals with PSD are at an increased risk of experiencing severe complications such as aspiration pneumonia, malnutrition, dehydration, and other conditions closely associated with prolonged hospital stays (Crary et al., 2013; Dziewas et al., 2021; Kelly et al., 2006; Martin‐Harris & Jones, 2008; Pisegna & Langmore, 2016).

Emerging evidence indicates that lesion locations of stroke might influence PSD (Galovic et al., 2013, 2016, 2017; Hope et al., 2013; Wilmskoetter et al., 2020). The medulla oblongata, in particular, plays a crucial role in the regulation of swallowing function (Flowers et al., 2011). Pathological changes in this region may lead to the pharyngeal residue (PR), vocal cord paralysis, dysarthria, and abnormal laryngeal elevation (Horner et al., 1991; Meng et al., 2000). Additionally, cortical and subcortical brain regions are also involved in the regulation of swallowing function. For instance, the sensorimotor, parietal‐temporal, and insular cortex are essential cortical areas related to laryngeal elevation and vestibular closure (Wilmskoetter et al., 2019). The insular cortex, often regarded as a central hub of the “swallowing network,” has been reportedly associated with poorer swallowing function (Galovic et al., 2017). Moreover, subcortical brain regions, including basal ganglia, internal capsule, periventricular white matter, and thalamus, may be linked to the oral propulsive stage of swallowing (Cola et al., 2010), while the superior corona radiata may play an important role in the swallowing function recovery (Galovic et al., 2017).

Nasogastric tube placement is commonly used as a temporary solution for feeding patients with PSD. It was reported that approximately one‐fourth of patients with brainstem pathology may be unlikely to resume oral intake at discharge (Horner et al., 1991; Meng et al., 2000), and stroke in the anterior insular cortex may lead to a longer tube feeding duration to ensure adequate nutrition (Galovic et al., 2017; Hota et al., 2021; Joundi et al., 2019; Wilmskoetter et al., 2019). Yet, the disadvantages of tube feeding are also apparent: long‐term tube feeding would most probably cause the loss of sensation, social disorder, and severe complications including aspiration pneumonia (Chen et al., 2015).

Videofluoroscopy swallowing study and fiberoptic endoscopic evaluation of swallowing (FEES) are considered gold standard methods for assessing PSD (Dziewas et al., 2021). FEES, in particular, is a sensitive tool used to evaluate the PR, aspiration, and anatomical structures of the nasopharynx and supraglottic area (Kelly et al., 2006; Martin‐Harris & Jones, 2008; Pisegna & Langmore, 2016). However, the specific characteristics of PSD associated with different stroke locations, such as supratentorial and infratentorial strokes, have not been extensively explored with FEES (Daniels et al., 2017; Moon et al., 2012). Therefore, the present study adopted FEES to investigate the characteristics of PSD caused by different lesion sites, and further explore factors influencing the duration of nasogastric tube placement.

## 2 MATERIALS AND METHODS

### 2.1 Study populations

From November 2018 to June 2021, stroke patients in the Department of Rehabilitation Medicine, the Third Hospital of Sun Yat‐sen University were screened for analysis. The clinical characteristics, including age, gender, stroke types, lesion sites of stroke, comorbidities, and swallowing function, were investigated. The possible dysphagia was initially evaluated by a well‐trained speech‐language pathologist (SLP) after admission, and the lesion sites of the stroke were confirmed by the radiologist. To investigate the factors influencing the need for tube feeding, we only included individuals with stroke and severe impairment of oral intake (Functional Oral Intake Scale [FOIS] score < 4) during the initial swallowing evaluation. The FOIS score was obtained through telephone follow‐up by the SLP within the first year after the onset of PSD. Ethical approval for this study was obtained from the Third Hospital of Sun Yat‐sen University (No. 02‐351‐01), and an exemption for informed consent was granted.

### 2.2 Inclusion criteria and excluded criteria

The inclusion criteria were: (1) age ≥ 18 years; (2) PSD was confirmed by FEES and clinical evaluations; (3) lesion sites of stroke were diagnosed by MRI, CT, and clinical symptoms; (4) stroke or PSD was confirmed limited in six months; (5) the lesion sites of stroke were limited in supratentorial or infratentorial lesions; and (6) the time interval between lesion sites of stroke and swallowing function evaluation was less than 2 weeks.

The excluded criteria were: (1) age < 18 years; (2) no impaired efficacy and/or safety during oropharyngeal swallowing was observed after FEES and clinical evaluations (Clavé et al., 2008); (3) a history of dysphagia caused by other neurological diseases or head and neck structural abnormality or damage; and (4) patients did not meet all inclusion criteria.

### 2.3 Swallowing function evaluation

The patients with suspicious dysphagia after stroke would undergo a series of standard evaluations to assess the swallowing function after admission. Dysphagia symptoms were initially assessed using the volume‐viscosity swallow test (V‐VST) and the Chinese eating assessment tool (EAT‐10). The volume‐viscosity swallow test and EAT‐10 were performed by an SLP, which are widely used questionnaires to assess the swallowing function in clinical practice (Belafsky et al., 2008; Riera et al., 2021). Symptomatic patients, defined as those with an EAT‐10 score of ≥3 or impaired safety and efficacy of swallowing, underwent a modified standardized FEES protocol. The FEES procedure was conducted by a well‐trained rehabilitation physician using a flexible endoscope mounted on an EndoSTROBE camera (ATMOS MedizinTechnik GmbH & Co.KG). FEES was conducted with colored (dyed with green) thickened liquid, and the viscosity of the liquid was categorized into four levels: lower than 50 mPa.s, in the range of 51–350 mPa.s, 351–1750 mPa.s, and above 1750 mPa.s (Calmarza‐Chueca et al., 2021). If no significant aspiration occurred with different viscosities of liquid, additional tests were conducted using solid consistencies such as mashed rice‐porridge, fruit jelly, bananas, and crackers (De Stefano et al., 2021; Pawlitzki et al., 2021; Pizzorni et al., 2021). A volume of 3, 5, and 10 mL of colored thickened liquid using a syringe and one teaspoon (about 3 g) of the other test foods was examined in order (Tsubokawa et al., 2022).

**FIGURE 1:**
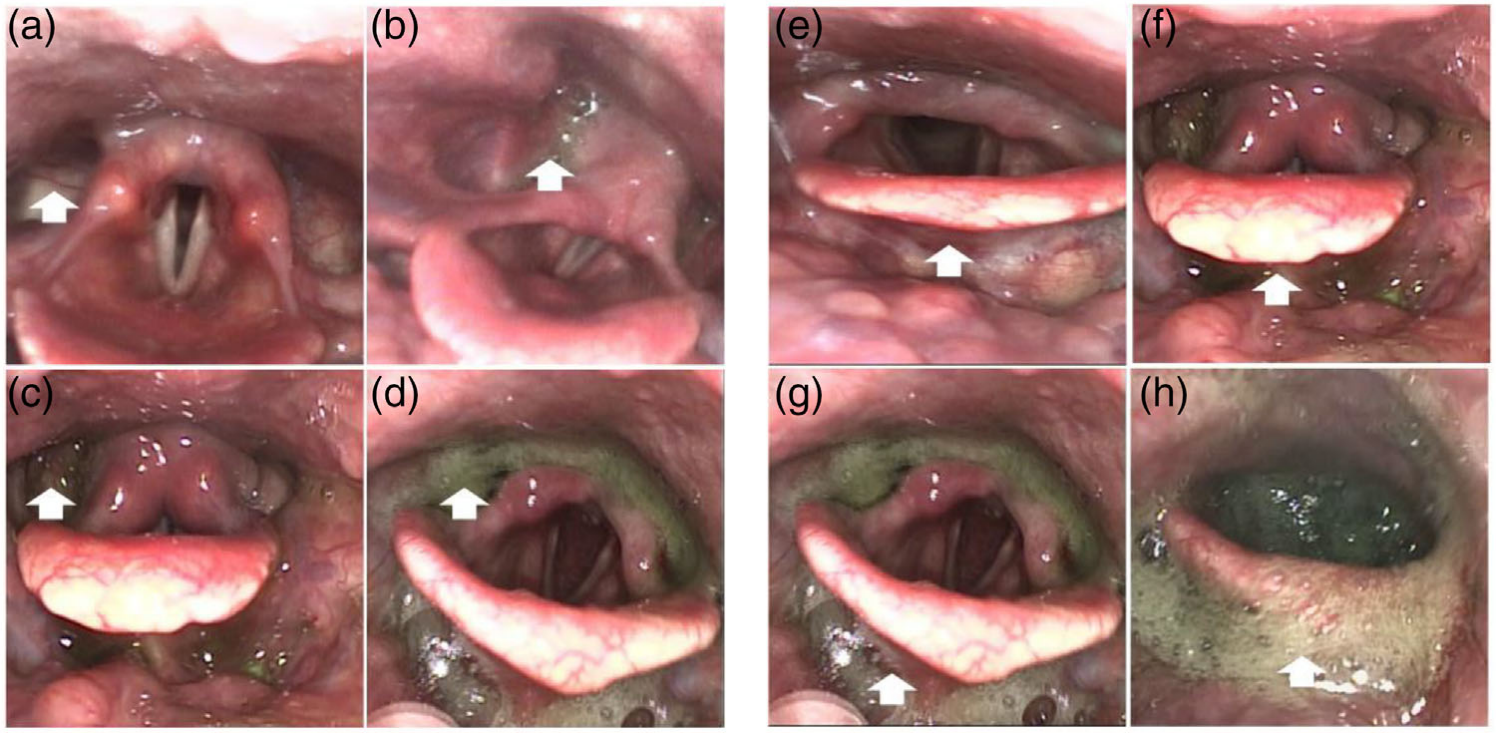
The pharyngeal residue from FEES examination. **(a‐d)** The residue in the pyriform sinus scale from 2 to 5; **(e‐h)** the residue in the valleculae scale from 2 to 5. *Note*: FEES, fiberoptic endoscopic evaluation of swallowing.

The penetration‐aspiration scale (PAS) was used to assess penetration and aspiration events, which were evaluated by the SLP. PAS is a validated ordinal scale ranging from 1 (no penetration aspiration) to 8 (silent aspiration), and the higher the score, the more severe the penetration and aspiration symptoms (Hamidon et al., 2006; Murray et al., 1996). The Yale PR Severity Rating Scale was utilized to evaluate the amount of post‐swallow residue in the valleculae and the pyriform sinus, which is a validated ordinal scale ranging from 1 (no residue) to 5 (severe residue) (Figure 1) (Pizzorni et al., 2021; Van den Berg et al., 2013). The highest PAS and PR scores recorded during the FEES examination were included in the final analysis of this study. In addition, the Murray Secretion Severity Rating Scale (MSS) was applied to evaluate the severity of accumulated oropharyngeal secretions (Tsubokawa et al., 2022). Laryngopharyngeal sensation (LS) was tested to predict dysphagia and identify patients at risk. The absence of laryngeal adductor reflex or cough after contact with at least one arytenoid was categorized as impaired LS in the present study (Shapira‐Galitz et al., 2019). The condition of vocal fold motion was also recorded, vocal fold motion impairment (VFMI) includes vocal fold immobility (no appreciable vocal fold movement on one side or both sides), vocal fold hypomobility, vocal fold paralysis, or vocal fold paresis was evaluated (Rosen et al., 2016). Nutritional intake was measured using the FOIS, a validated ordinal scale ranging from 1 to 7. In this study, time‐to‐event endpoints (oral feeding) referred to FOIS ≥ 4 (Souza et al., 2020; Sura et al., 2020). Besides, the Modified Rankin Scale (mRS) was used to assess their functional status after stroke. Two investigators (FS and JQ) collaborated on data extraction, and any inconsistencies or errors in the records were double‐checked and proofread.

### 2.4 Brain lesions evaluation

The stroke types and lesion sites were confirmed by MRI, CT, and clinical symptoms. Both hemorrhagic stroke and ischemic stroke were included in the present study. The lesion sites were assessed by a well‐trained radiologist, and only patients with lesion sites limited in supratentorial or infratentorial ischemic and hemorrhagic stroke were included. According to the lesion sites, the included patients were further categorized into the supratentorial group (including lobar and deep intracerebral stroke subgroups) and infratentorial stroke group (including brainstem and cerebellar stroke subgroups).

### 2.5 Statistical analysis

The Shapiro‐Wilk test and Levene's test were used to test the normality and homogeneity. Continuous variables were presented as mean ± standard deviation (SD), while categorical variables were presented as frequencies. The clinical characteristics, including gender, stroke types, and comorbidities, were reported as participant numbers and analyzed using the Pearson chi‐square test (χ2) or Fisher's exact test. Continuous variables of the baseline characteristics between groups were analyzed using the two‐independent sample *t*‐test or Wilcoxon rank‐sum test (as appropriate). The Mann‐Whitney U test or Pearson chi‐square test (χ2) was used to test the differences including PAS, PR, MSS, LS, and the condition of the vocal fold between groups. Time‐to‐event endpoints (oral feeding) after PSD were assessed using Kaplan‐Meier analysis stratified by lesion sites, PAS scores, PR scores, the condition of the vocal fold, mRS score, and the log‐rank test was used to compare the patterns of cumulative indwelling nasogastric tube rates. Statistical analysis was performed using SPSS software (version 20.0, IBM), and statistical charts were finished with the use of GraphPad Prism (version 9.0, GraphPad Software Inc.). *p* values less than 0.05 were considered to indicate statistical significance.

## 3 RESULTS

### 3.1 The general characteristics of patients

A total of 94 PSD patients were included in the final analysis, among them, 40 patients had supratentorial strokes (including 7 lobar strokes, 16 deep intracerebral strokes, and 17 patients with both lobar and deep intracerebral involvement), while 54 patients had infratentorial strokes (including 36 brainstem strokes, 3 cerebellar strokes, and 15 patients with both brainstem and cerebellar involvement).

In the supratentorial and infratentorial stroke groups, the mean age (±SD) were 63.86 (±12.97) and 58.69 (±10.66) respectively (*p* = .37); the percentage of males was 82.5 and 70.4%, respectively (*p =* .176). A total of 42.5 and 66.7% patients were diagnosed for ischemic stroke in the two groups (*p* = .020). A significant difference was found in tracheostomy between the two groups (*p* = .043), while no significant differences were found in hypertension, hyperlipidemia, diabetes, and pneumonia (*p* > .05) (Table 1).

**TABLE 1:** General characteristics of patients.

Variables	Supratentorial stroke (n = 40)	Infratentorial stroke (n = 54)	*p‐value*
Age mean (yr) (SD)	63.86 (12.97)	58.69 (10.66)	.370
Gender n (%)			
Male	33 (82.5)	38 (70.4)	.176
Stroke types n (%)			
Ischemic	17 (42.5)	36 (66.7)	.020*
Hemorrhagic	23 (57.5)	18 (33.3)	
Comorbidities n (%)			
Hypertension	32 (80)	36 (66.7)	.153
Hyperlipidemia	8 (20)	8 (14.8)	.508
Diabetes	14 (35)	14 (25.9)	.342
Tracheostomy	11(27.5)	19 (35.9)	.043*
Pneumonia	30 (75)	38 (70.4)	.062

*Note*:*p < .05.

### 3.2 The characteristics of PSD between supratentorial and infratentorial stroke groups

**FIGURE 2:**
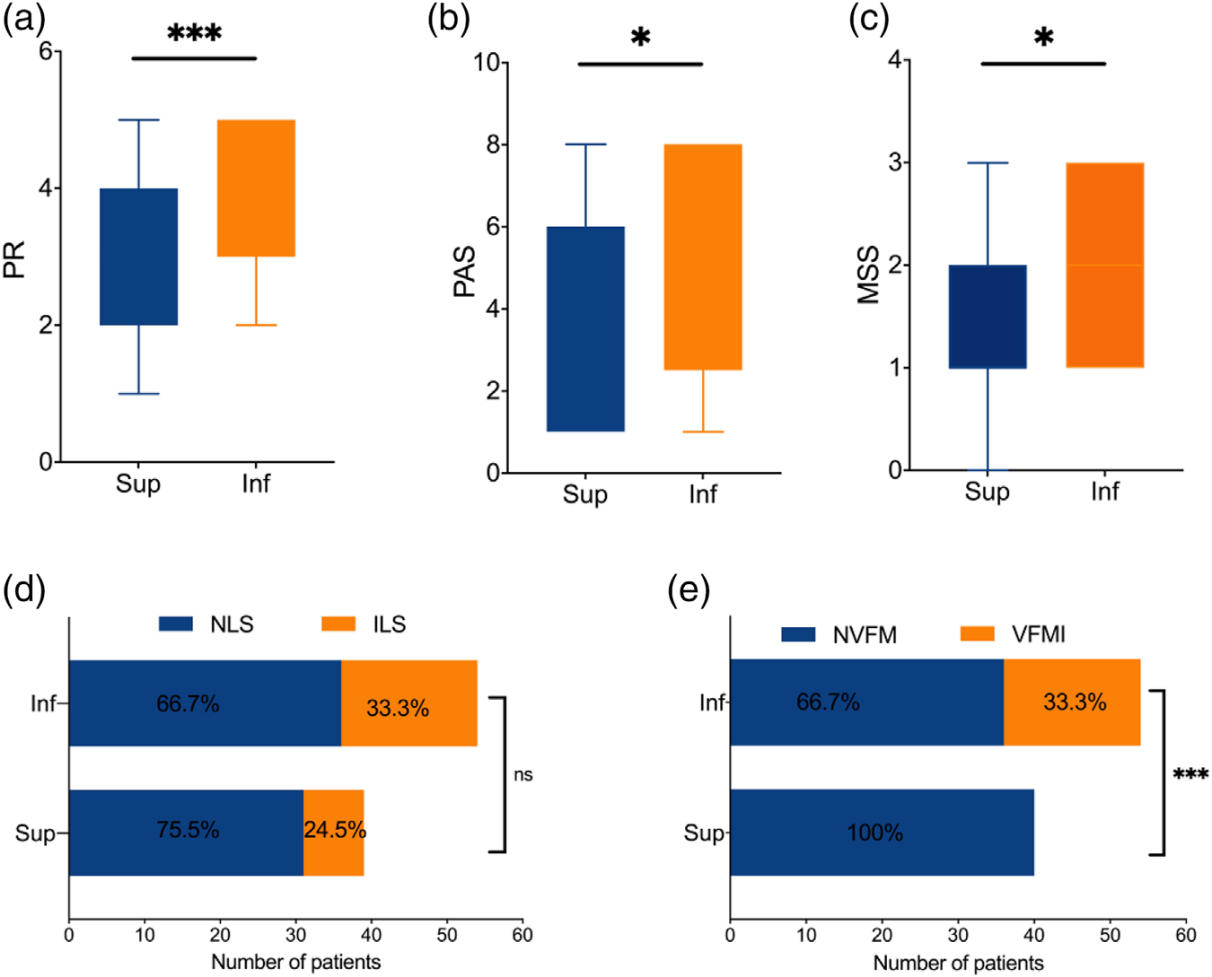
The comparison of pharyngeal residue severity rating scale (PR), penetration‐aspiration scale (PAS), Murray secretion severity rating scale (MSS), laryngopharyngeal sensation (LS), and the condition of vocal fold between groups stratified by supratentorial or infratentorial stroke. The PR scores **(A,** *p* < .001), PAS scores **(B,** *p* < .05), and Murray secretion severity rating scale scores **(C,** *p* < .05) in the infratentorial stroke group were significantly higher than that in the supratentorial stroke group. **(D)** There was no difference in LS between groups (*p* > .05). **(E)** The occurrence of vocal fold immobility in the infratentorial stroke group was significantly higher than that in the supratentorial stroke group (*p* < .001). *Note*: PR, pharyngeal residue severity rating scale; PAS, penetration‐aspiration scale; MSS, Murray secretion severity rating scale; LS, laryngopharyngeal sensation; NLS, normal laryngopharyngeal sensation; ILS, impaired laryngopharyngeal sensation; NVFM, normal vocal fold mobility; VFMI, vocal fold motion impairment; Inf, infratentorial stroke; Sup, supratentorial stroke; *p < .05, ***p < .001, ns, no significance.

In the infratentorial stroke group, the PR scores (*p* < .001), PAS scores (*p* < .05), and MSS scores (*p* < .05) were significantly higher compared to the supratentorial stroke group (Figure 2a‐c). The worse vocal fold mobility was also observed in the infratentorial stroke group (*p* < .001) (Figure 2e), which suggested that the infratentorial stroke might lead to worse swallowing function as compared to supratentorial stroke. However, no significant difference was observed in the LS between the two groups (Figure 2d).

### 3.3 The characteristics of PSD among lobar, deep intracerebral, brainstem, and cerebellum stroke subgroups

**FIGURE 3:**
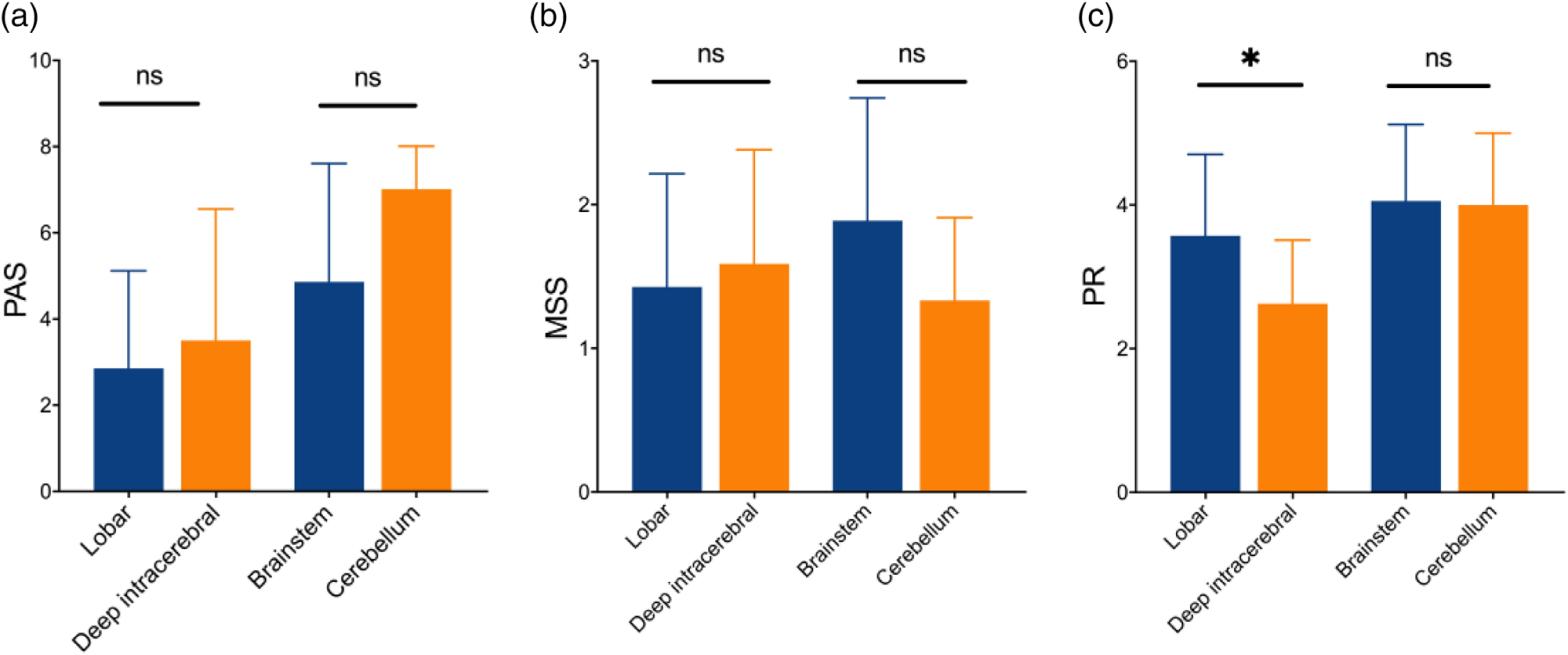
The comparison of PAS, MSS, and PR scores stratified by lobar, deep intracerebral, brainstem, and cerebellum stroke. There was no difference in PAS scores **(a)** and Murray secretion severity rating scale scores **(b)** between subgroups (*p* > .05). The PR score was significantly higher in lobar stroke than deep intracerebral subgroup (*p* < .05), while no difference was observed between brainstem and cerebellum stroke subgroups (*p* > .05) **(c)**. *Note*: PAS, penetration‐aspiration scale; MSS, Murray secretion severity rating scale; PR, pharyngeal residue severity rating scale; **p < .05, ns, no significance.

As shown in Figure 3, the PR scores in the lobar stroke subgroup were significantly higher as compared to the deep intracerebral stroke subgroup (*p* < .05) (Figure 3c), while no differences were found in PAS scores and MSS scores between the lobar and deep intracerebral stroke subgroups (*p* > .05) (Figure 3a and c), indicating that the lobar stroke might result in more severe impairment of swallowing function compared to deep intracerebral stroke. Furthermore, no differences were found in PAS scores, PR scores, and MSS scores between the brainstem and cerebellum stroke subgroups (*p* > .05) (Figure 3a‐c).

### 3.4 Factors that influence the time before oral feeding after PSD

**FIGURE 4:**
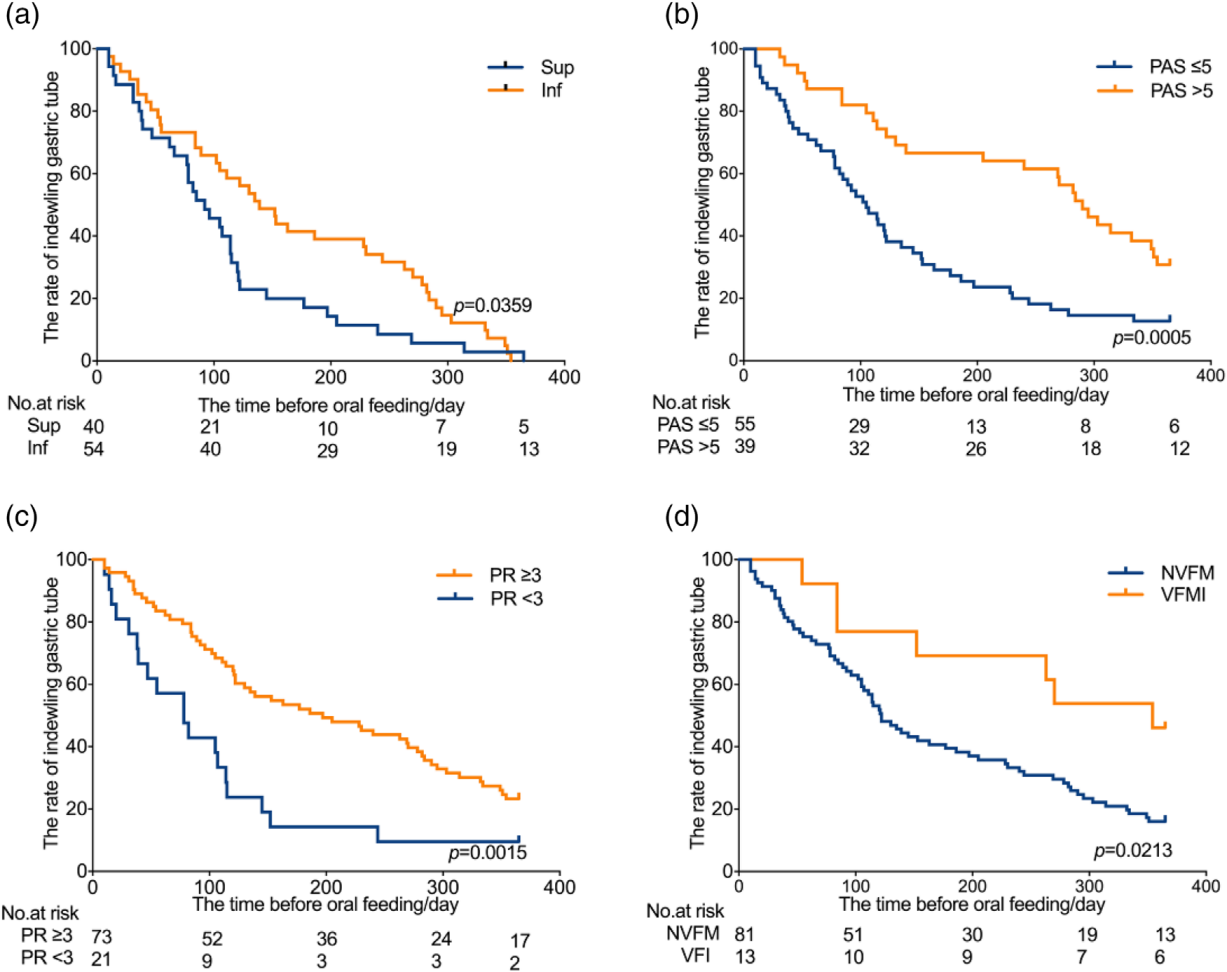
Kaplan‐Meier analysis for time‐to‐event end points (oral feeding) stratified by supratentorial or infratentorial stroke, PAS scores, PR scores, and the condition of vocal fold. A significant difference was found in supratentorial and infratentorial stroke groups **(a**, *p* = .0346), and there were significant differences between PAS > 5 versus PAS ≤ 5 **(b**, *p* = .0005), PR ≥ 3 verus PR < 3 **(c,** *p* = .015), normal vocal fold mobility versus vocal fold immobility groups **(d**, *p* = .0213). *Note*: PAS, penetration‐aspiration scale; PR, pharyngeal residue severity rating scale; NVFM, normal vocal fold mobility; VFMI, vocal fold motion impairment.

Kaplan‐Meier survival analysis was conducted to assess the duration of nasogastric tube dependence following PSD based on various factors (including stroke lesion sites, PAS scores, PR scores, the condition of vocal fold, and mRS scores). The mean time to oral feeding was 92 days in the supratentorial stroke group, and 139 days in the infratentorial stroke group. A significant difference was found in the two groups (*p* = .0359, log‐rank test) (Figure 4a). Moreover, there were significant differences in time‐to‐oral feeding between PAS > 5 versus PAS ≤ 5 (*p* = .0005, log‐rank test), PR ≥ 3 versus PR < 3 (*p* = .015, log‐rank test), normal vocal fold mobility versus VFMI groups (*p* = .0213, log‐rank test) (Figure 4). However, no significant difference was found between mRS < 3 and mRS ≥ 3 groups (*p* = .1591, log‐rank test). These results demonstrated that the stroke lesion sites, PAS scores, PR scores, and vocal fold mobility might influence the time required before resuming oral feeding after PSD.

## 4 DISCUSSION

The main findings of the present study were: (i) Infratentorial stroke patients exhibited worse swallowing dysfunction compared to the supratentorial stroke group, which manifested as higher PR, PAS, MSS scores, and worse vocal fold mobility. (ii) The lobar stroke patients may lead to worse swallowing function as compared to the deep intracerebral stroke group, which manifested as higher PR scores. (iii) The stroke lesion sites, PAS scores, PR scores, and vocal fold mobility might be associated with the duration of nasogastric tube dependency after PSD.

### 4.1 The characteristics of PSD among different lesion sites after stroke

The present study's findings suggest that swallowing function is more adversely affected in patients with infratentorial stroke as compared to supratentorial stroke. The infratentorial brain regions, including the pons and medulla, were identified as a center in the regulation of swallowing, which participated in the coordinated contraction of lingual, pharyngeal, and laryngeal muscles (Horton et al., 2018; Ko et al., 2021; Meng et al., 2000; Smith et al., 2013). In theory, any damage to this center may result in dysphagia. For example, some evidence has suggested that medullary infarctions are associated with issues like upper esophageal sphincter (UES, a piece of muscle that will quickly open and contract when swallowing) dysfunction and pharyngeal weakness (Ko et al., 2021; Meng et al., 2000). Moreover, higher PR and MSS scores were also observed in the infratentorial stroke group, which further indicated the importance of infratentorial brain regions. The accumulation of saliva was reported to correlate with reduced laryngeal sensation, laryngeal displacement, and UES relaxation, all of which were modulated by the infratentorial brain regions (Borders et al., 2019; Brady et al., 2016; Yamaguchi et al., 2019). Furthermore, some anatomical reasons may also be important. For example, the superior laryngeal nerve is responsible for the laryngeal adductor response and airway protection. Some peripheral signals collected by it will be transited to the central nervous system through the solitary tract nucleus in the brainstem (Foote & Thibeault, 2021). Thus, patients with pathological changes in the brainstem may be more susceptible to aspiration and vocal fold immobility.

Interestingly, the lobar stroke may also lead to worse swallowing function as compared to the deep intracerebral stroke according to the results of our study. This finding indicated that, in addition to the medulla oblongata, the cortical and subcortical regions also play an important role in regulating swallowing function. Lesions in specific areas, such as the supramarginal gyrus, angular gyrus, parietal‐temporal cortical regions, and postcentral gyrus, have been reportedly associated with oropharyngeal residue, indicating their involvement in swallowing function (Suntrup‐Krueger et al., 2017; Wilmskoetter et al., 2019). The postcentral gyrus, in particular, serves as a central hub for integrating sensorimotor information and plays a role in processing swallowing signals from the brainstem. Damage to the postcentral gyrus can disrupt afferent sensory input from the oropharynx, leading to oropharyngeal residue (Dziewas et al., 2003).

### 4.2 Factors that influence the need for tube feeding after PSD

The findings of the present study suggest that stroke lesion sites, PAS scores, PR scores, and vocal fold mobility might influence the time required for oral feeding after PSD. First, patients with infratentorial stroke may experience a longer duration before transitioning to oral feeding. This is likely due to the observation that individuals with infratentorial stroke exhibit worse swallowing function, as mentioned earlier. Second, patients with higher PAS scores, which indicate a greater risk of aspiration, are associated with a greater reliance on tube feeding. Aspiration, the entry of food or gastric contents into the respiratory tract during or after swallowing, poses significant risks such as aspiration pneumonia (Alhashemi, 2010; Hansen et al., 2008; Macht et al., 2011; Martino et al., 2005; Son et al., 2017). Hence, patients with higher PAS scores require tube feeding to prevent severe complications. Third, higher PR scores, reflecting impaired pharyngeal bolus clearance (Rademaker et al., 1994), may prolong the need for tube feeding. PR occurs when food particles are not adequately cleared from the pharynx due to factors such as impaired UES opening, weak pharyngeal constrictor muscles, or weak lingual muscles (Dejaeger et al., 1997; Kim et al., 2015). Increased PR raises the risk of aspiration, necessitating a longer duration of nasogastric tube placement (Molfenter & Steele, 2013; Shapira‐Galitz et al., 2019). Last, patients with vocal fold immobility may require an extended period of nasogastric tube feeding after PSD. The pharynx is considered the intersection of the upper respiratory and digestive tract, which connects the nasal cavity, oral cavity, larynx, and esophagus (Ong & Steele, 2018). Pharyngeal swallowing is a complex biomechanical process that converts the respiratory tract into the swallowing tract within 500 ms to transport food particles into the pharynx and UES safely (Tran et al., 2018), in which the vocal fold mobility plays a crucial role. Vocal fold immobility can lead to an increased risk of aspiration, necessitating the use of a nasal tube.

These findings demonstrated that a comprehensive assessment is necessary for patients with infratentorial stroke, higher PAS scores, higher PR scores, and vocal fold immobility before the removal of nasogastric tube to avoid complications after PSD. Effective preventive and treatment measures are required to shorten the transition to oral feeding. For example, foods with a certain viscosity are recommended for patients with higher PAS scores to minimize the risk of aspiration (McCarty & Chao, 2021). Additionally, patients with a decline in UES opening, usually indicated by higher PR scores, may benefit from interventions such as balloon dilatation and botulinum toxin injection (Xie et al., 2022). For patients with vocal fold immobility, rehabilitative strategies, including posture techniques and swallowing maneuvers, are recommended; while for those who do not benefit from rehabilitative strategies, surgical treatment may be more suitable including injection laryngoplasty and medialization thyroplasty (Giraldez‐Rodriguez & Johns, 2013).

## 5 LIMITATIONS

Our study has several limitations. First, during the FEES examination, the “whiteout” may have affected the accuracy of aspiration assessment, which was a transient white endoscopic picture during the pharyngeal contraction. Therefore, a comprehensive assessment, including videofluoroscopy swallowing study, is further required. Second, the LS was evaluated by using the endoscopic contact with the arytenoid, which may have been influenced by operator variability. Third, the time elapsed from stroke onset to FEES examination was not strictly limited, which may lead to potential bias in the results. Fourth, the detailed information on stroke, including lesion size, side of the lesion, and history of thrombolytic therapy, was not well described in our study due to the limited data. The prospective studies are urgently needed to verify our results.

## 6 CONCLUSIONS

Infratentorial stroke may contribute to a poorer swallowing function as compared to supratentorial stroke, which manifests as a higher PR, PAS, and MSS scores and worse vocal fold mobility. Furthermore, patients with infratentorial stroke, higher PAS scores, higher PR scores, or VFMI may require a longer duration of nasogastric tube placement. Early and appropriate preventive treatment measures are essential to improving the swallowing function of these patients and shortening the transition to oral feeding.

## CONFLICTS OF INTEREST STATEMENT

The authors have no funding or conflict of interest.

### PEER REVIEW

The peer review history for this article is available at https://publons.com/publon/10.1002/brb3.3161

## Data Availability

The data that support the findings of this study are available from the corresponding author upon reasonable request.
